# Long unidirectional barbed suturing technique with extracorporeal traction in laparoscopic myomectomy

**DOI:** 10.1186/1471-2482-14-84

**Published:** 2014-10-27

**Authors:** Yoichi Aoki, Iwaho Kikuchi, Jun Kumakiri, Mari Kitade, Azusa Shinjo, Rie Ozaki, Yu Kawasaki, Satoru Takeda

**Affiliations:** Department of Obstetrics and Gynecology, Juntendo University School of Medicine, Hongo 2-1-1, Bunkyo-ku, Tokyo, 113-8421 Japan

## Abstract

**Background:**

Myomectomy is now often performed laparoscopically rather than by laparotomy to alleviate the risk of postoperative adhesions and reduce postoperative pain. However, intracorporeal knot-tying under direct laparoscopy is difficult and requires proficiency. We conducted a retrospective study comparing the results of a long unidirectional barbed suturing technique (with V-Loc180 suture) and the results of conventional suturing as applied to laparoscopic myomectomy.

**Methods:**

In women who underwent laparoscopic myomectomy in our university hospital between January 2011 and April 2013, uninterrupted suturing of 2 or more layers was performed. These women were divided into 2 groups according to the method of suturing: those in whom standard absorbable sutures were used (group P, n =42) and those in whom our suturing technique was used (group V, n =41). Patient characteristics and surgical variables were compared between the 2 groups.

**Results:**

No significant between-group difference was observed in age (p = .975), body mass index (p = .778), GnRHa administration (p = .059), intraoperative vasopressin dose (p = .364), intraoperative blood loss (73.8 ± 64.1 vs. 59.3 ± 54.0 mL, respectively; p = .199), myoma mass (212.6 ± 133.3 vs. 208.3 ± 198.4 g, respectively; p = .134), ΔHb (p = .517), or postoperative hospital stay (p = .314). Operation time (mean ± SD) was significantly shorter for group V (71.2 ± 22.9 minutes; range, 28.0–110.0 minutes; p < .001) than for group P (94.4 ± 27.2 minutes; range, 53.0–165.0 minutes). No patient required intraoperative transfusion or conversion to laparotomy.

**Conclusions:**

Our suturing technique exploits the features of unidirectional barbed sutures and can be used in the same way as the conventional method when performing continuous suturing for laparoscopic myomectomy. Our data suggest that operation time can be reduced by as much as 25% with this new technique.

**Electronic supplementary material:**

The online version of this article (doi:10.1186/1471-2482-14-84) contains supplementary material, which is available to authorized users.

## Background

Uterine myoma develops in approximately 25% of premenopausal women [1, 2] and can lead to dysmenorrhea, hypermenorrhea, infertility, and other disorders. For symptomatic women who do not desire children, hysterectomy and uterine artery embolization are common treatment options; however, most symptomatic women who desire children opt for myomectomy. Myomectomy is now often performed laparoscopically [3, 4] rather than by laparotomy to alleviate the risk of postoperative adhesions [5–7] and reduce postoperative pain.

Laparoscopic myomectomy (LM) requires suturing of the muscularis propria after enucleation of the myoma to prevent a myometrial defect. Currently, absorbable suture material is used mainly for interrupted or continuous suturing to repair areas in which the muscular layer has been lost. However, intracorporeal knot-tying under direct laparoscopy is difficult and requires proficiency. We have closed more than 2 layers of the myometrium with continuous sutures and intracorporeal knots by close coordination between the operating surgeon and an assistant. To reduce blood loss from the myomectomy site(s), gonadotropin releasing hormone analogue (GnRHa) [8] and vasopressin diluted in 100 mL of saline are administered to patients preoperatively by local uterine injection [9].

Barbed sutures have been recently introduced for suturing subcutaneous tissue and for layers that require continuous suture, such as the myometrium. The main difference between barbed sutures and conventional absorbable sutures is that the former have barbs that will not loosen their grip once tension is applied [10]. We developed a suturing and tensing technique that takes advantage of the barbed sutures, and we conducted a retrospective study to compare the surgical results of our technique with those of the conventional method. Our technique is described herein as well as the results of our study.

## Methods

### Patients

Eighty-three patients who underwent LM for one or more intramural myomas were included in the present study. These patients were identified from among 818 patients who underwent uterine LM at our institution between January 2011 and April 2013, with 309 of these patients treated by a certified endoscopic surgeon (I.K.) who has performed over 3000 laparoscopic surgeries in Japan. No patient who had undergone a concomitant procedure, such as removal of the ovaries or fallopian tubes, or who had undergone adhesiolysis for severe adhesions was included in the study.

All 83 patients had undergone magnetic resonance imaging before surgery to confirm that the tumors were not malignancies. All patients had provided informed consent for LM, for the operative procedures to be recorded on video, and for their clinical data to be used for research purposes, with the understanding that the data would be anonymized. The study, which was in full compliance with the Declaration of Helsinki, was approved by the ethics committee of Juntendo University Hospital.

We began using the barbed suture technique in January 2012 and were thus able to compare 2 large groups of patients: those treated between January 2011 and December 2011 in whom conventional suturing was performed and those treated between January 2012 and April 2013 in whom the barbed suturing technique was performed. Nearly all patients were given 1.88 mg of gonadotropin-releasing hormone (GnRH) agonist leuprolide acetate (leuprorelin, Takeda Pharmaceuticals, Osaka, Japan) before surgery.

### Surgical procedure

LM was performed as previously described [11]. Briefly, we applied the 4-puncture technique. Vasopressin (20 IU), diluted in 100 mL of saline, was infused into the myoma between the capsule and normal muscle layer, and a horizontal incision was made just above the myoma with the use of monopolar forceps. The myoma was extracted with a myoma screw, and a 2–5-layer running suture was used to close the myometrium.

In the first group of patients (group P, n =42), we used 0-Polysorb suture with a 1/2 37-mm curved needle (Covidien, Mansfield, MA, USA) (Group P, n =42). In the second group of patients (group V, n =41), we used 1 piece of unidirectional barbed 0-V-Loc 45-cm TM 180 suture with a single 1/2 37-mm curved needle (Covidien) in cases of a single myoma or, in cases of multiple myomas, 1 piece of 0-V-Loc 45-cm (VLOCL0326) for suturing the largest myomectomy wound and conventional 0-Polysorb suture for the other wounds. With the conventional method, the number of suture layers is dictated by the depth of the uterine defect, and the sutures are placed perpendicular to the incision. Closure of the serosal layer and myometrium is performed by continuous suturing with 0-Polysorb suture. All suturing was performed intracorporeally (see Additional file 1).

The enucleated myomas were removed with the aid of an electromechanical morcellator (Ethicon Inc., Somerville, NJ, USA). Finally, depending on the wound characteristics, the sutured surface was covered with GYNECARE INTERCEED Absorbable Adhesion Barrier (Ethicon, Inc.).

### Technique for unidirectional barbed suturing

**Figure 1:**
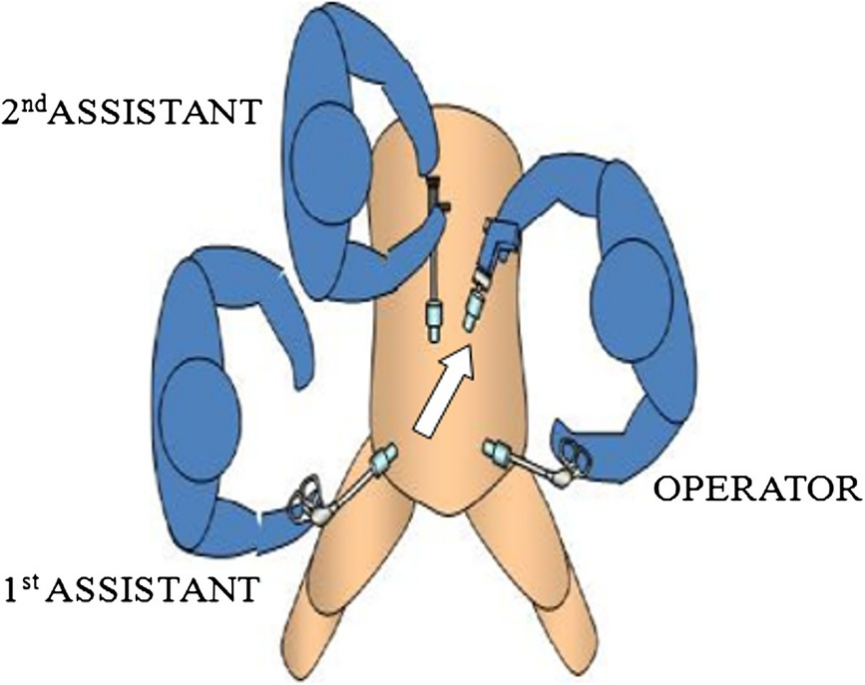
**Direction in which the needle is drawn.**

With use of 0-V-Loc 45-cm TM 180 suture thread, the first tie was secured by passing the needle through the tail loop. We stopped the first suture before the muscle defect, drew the thread outside the body with needle forceps, grabbed hold of the tail loop that had been held outside the body with needle forceps, and passed it over the needle that was still in the muscle defect (Additional file 2). The first layer was then sutured from right to left, and when the needle reached the wound edge, it was turned in the reverse direction, and the second layer was sutured from left to right. The suture thread was 45 cm in length. After 1 suture was complete, it was tensed through a 12-mm trocar by drawing the needle outside the body cavity in the direction indicated in Figure 1 (Additional file 3). Most of the 0-Polysorb continuous sutures in the serosal layer were interlocked, but the 0-V-Loc TM 180 barbed suture in the serosal layer was completed as a baseball suture (Additional file 4) so that the barbs were not exposed. This was done to avoid the risk of damage to other organs. Finally, the muscular layer was massaged, and the suture thread was cut as close to the wound as possible while the tautness was maintained. The end of the suture thread was prevented from protruding from the wound site (Additional file 5).

### Variables compared

The following variables were compared between the 2 groups of patients: age; body mass index (BMI); doses of GnRHa and vasopressin; intraoperative blood loss, which was calculated by subtracting the amount of physiological saline used for peritoneal washing from the total amount of fluid removed via suction; operation time, which was taken as the time from the first incision (initial insertion of the trocar) to the time when the trocar incision was closed (and thus the surgery was completed); postoperative change in hemoglobin (ΔHb), which was determined by subtracting the Hb (g/dL) measured on postoperative day 1 from that measured within 1 month prior to surgery, myoma mass, and length of postoperative stay.

### Statistical analysis

Values are shown as mean ± SD and range. Between-group differences were analyzed by Student’s *t*-test or Mann–Whitney U-test, and significance was accepted at a probability (p) value of < .05. All statistical analyses were performed with SPSS v20 statistical software (IBM, Tokyo, Japan).

## Results

Results of the study are shown in Table 1. No significant difference in patient age was noted between group P and group V, nor was there a significant difference in BMI, preoperative GnRHa use, intraoperative vasopressin dose, intraoperative blood loss, ΔHb, myoma mass, or length of postoperative stay.

**Table 1:** **Clinical characteristics and results of laparoscopic myomectomy in our 2 study groups**

Variable	Group V (n = 41)	Group P (n = 42)	p value
Age (years)	37.4 ± 4.8	37.5 ± 5.2	.975
Body mass index (kg/m^2^)	22.06 ± 3.71	21.99 ± 3.38	.778
Preoperative GnRHa (no. of injections)	4.56 ± 1.43	5.11 ± 1.11	.059
Total operation time (minutes)	71.2 ± 22.9	94.4 ± 27.2	<.001
Myoma weight (g)	208.3 ± 198.4	212.6 ± 133.3	.134
Intraoperative blood loss (mL)	59.3 ± 54.0	73.8 ± 64.2	.199
Change in hemoglobin (g/dL)	1.27 ± 0.88	1.37 ± 0.83	.517
Hospital stay (no. of days)	3.00 ± 0.00	2.97 ± 0.41	.314
Vasopressin (IU)	6.00 ± 2.38	6.79 ± 3.12	.364

Group V = patients in whom V-Loc suture was used, Group P = patients in whom 0-Polysorb suture was used, GnRHa = gonadotropin releasing hormone agonist.

Operation time was significantly shorter in group V than in group P (71.2 ± 22.9 minutes; range, 28.0 – 110.0 minutes vs. 94.4 ± 27.2; range, 53.0 – 165.0 minutes; p < .001).

All surgeries were performed under general anesthesia. There was no damage to other organs and no intraoperative complication. No patient required intraoperative transfusion or conversion to laparotomy. Surgical pathology-based diagnosis was uterine leiomyoma in all cases. All patients were examined postoperatively at 1, 3 and 6 months, and there were no complications.

## Discussion

Our study, which compared barbed suturing with conventional suturing for uterine leiomyoma, showed that the suturing technique applying V-Loc suture material reduced operation time by approximately 25%. Although laparoscopic suturing of the uterine myometrial wall is difficult and proficiency is required, previous clinical studies have indicated that the use of barbed suture reduces the time spent on suturing [12, 13], and this was confirmed in an animal model [14]. A possible explanation for the reduction in operation time is that because of the barbs, once the suture has been pulled taut, the points of commissure will not loosen even if the assistant does not maintain tension on the suture thread. In addition, with our technique, tension is applied in only 1 direction, toward the abdominal wall, thereby fixing the barbs away from digestive tract, greater omentum, and other structures. Also, to gain distance for pulling the 45-cm long barbed suture, we thought it effective to pull it through a trocar out of the abdominal cavity. Furthermore, this technique allows suturing of up to 3 layers. Alessandri et al. [12] performed continuous unidirectional barbed suturing of only 1 layer and suggested the possibility of suturing 2 layers, whereas we used unidirectional barbed suture, which allowed continuous suturing of 2–3 layers. Einarsson et al. [15] performed 2-layer suturing of the uterine defect using bidirectional barbed suture.

The suturing method we used reduced the knot-tying by 4–6 times over the number required for the conventional method. Angioli et al. [13] reported that passing the needle through the loop of the V-Loc suture requires great proficiency but that it can accelerate the suturing process. However, as we showed, the needle can be easily passed through the loop, and this hastens the work and improves stability (Additional file 2). Also, unlike conventional continuous sutures, barbed sutures do not require an assistant to apply tension to the suture thread, which may also contribute to a reduction in suturing time.

A review of reported studies (Table 2) revealed that Alessandri et al. [12] were not able to significantly reduce operation time with the use of V-Loc sutures; however, they were able to reduce the time required for suturing the uterine myometrial layer. Other reports indicated that blood loss was significantly reduced [12, 13].

**Table 2:** **Reported results of barbed suturing for myomectomy**

	CTF	Suture	No. of barbed suture cases	No. of control cases	Operation time (suture time) p value	Blood loss p value	Hospital stay p value	Vaso	GnRHa
Our study	II-2	V	41	42	<.001	.199	.314	Yes	Yes
Alessandri F et al. [12]	I	V	22	22	.177<.001	.004	-	No	No
Einarsson JI et al. [15]	II-2	B	107	31	.003	.76	.001	Yes	No
Angioli R et al. [13]	II-1	V	19	20	.062<.001	.008	.070	No	No
Ming-Chao Huang et al. [16]	II-1	V	34	34	<.001	.108	.486	No	No

CTF = Canadian Task Force classification; Vaso = vasopressin; GnRHa = gonadotropin releasing hormone agonist; V = unidirectional barbed suture (V-LOC180); B = bidirectional barbed suture.

Angioli et al. [13] were able to reduce both the time required for suturing and blood loss, whereas Huang et al. [16] showed that the use of unidirectional barbed suture reduced overall myomectomy time. Einarsson et al. [15] assessed the use of other types of barbed suture. There are indications that the use of barbed sutures shortens the postoperative hospitalization period. We found no between-group difference in postoperative stay, probably because even in cases in which no complication developed during the hospital stay, the number of days until discharge was fixed, per Japanese guidelines.

We found no significant between-group difference in blood loss; this was also true in the Einarsson et al. [15] study. Vasopressin was administered to our patients and to the Einarsson et al. patients. Thus, it is likely that the myometrial administration of vasopressin contributes more to reducing intraoperative blood loss than does decreased suturing time.

Greenberg et al. [10] reported the use of barbed suture for the first time in total laparoscopic hysterectomy, and its usefulness in closing the vaginal stump was discussed. In another study of barbed suture, the incidence of postoperative dehiscence was substantially reduced [17]. When residents and fellows performed the closure, barbed suture did not necessarily shorten the operation time relative to that taken by an experienced doctor using conventional suturing, although the suture time itself was significantly short [18]. Although Bogliolo et al. [19] reported significant shortening of the operation time with barbed suture, the incidence of postoperative complications, such as dehiscence and bleeding, did not differ significantly. In another study, only post-operative vaginal bleeding was significantly reduced [20].

Other studies have indicated that barbs can cause small bowel injury [21] and that within 30 days after total laparoscopic hysterectomy, small bowel obstruction can develop [22], possibly because the laparoscope can disrupt the suture line, allowing the barbs to get caught in the digestive tract and greater omentum. In addition, barbs left exposed outside the uterus after the suture thread is cut can become entangled in the digestive tract and cause small bowel obstruction, especially when the suture thread is tensed before being cut, drawing the thread into the myometrium. When continuous suturing is performed within a body cavity, the suture thread should be pulled taut frequently, especially when the work is performed within the restricted field of view typical of laparoscopic surgery, because the barbs frequently come into contact with other organs. The technique we followed is characterized by frequent advancement of the suture between the uterus and the trocar, minimizing contact between the barbs and other organs. Because the thread is drawn in only 1 direction when it is pulled taut to prevent contact with the digestive tract and the greater omentum, the thread should be grasped within 2 cm of the needle, where there are no barbs. Thus, by tensing the thread at that location and moving it back and forth, the thread can be drawn in 1 direction.

Because myomectomy is commonly performed in patients who desire children, it is important to preserve the patient’s ability to become pregnant and safely give birth; therefore, the possibility of vaginal delivery [23] should be considered. Because barbed sutures have been developed only recently, there are no reported studies of post-LM parturient outcomes. Therefore, future studies to assess the utility of barbed sutures in preserving natural birth are warranted.

Barbed sutures are more expensive than absorbable sutures. In Japan, the cost is approximately 10 times that of Polysorb sutures; thus, the cost-benefit of using a large number of barbed sutures is poor. However, the use of barbed suture reduces operation time by approximately 25%; thus, the time in the operating room and personnel costs are reduced.

We found surgery time to be greatly reduced with the suturing technique we describe. However, because of the retrospective nature of the study, the fact that there were slight differences in patient backgrounds between the 2 groups, and the fact that the technique of only 1 surgeon at 1 medical facility was evaluated, it is difficult to generalize our study findings. There is, therefore, a need for randomized controlled trials.

## Conclusions

Our suturing technique takes advantage of the particular benefits of long unidirectional barbed suturing and, like the conventional method, can be used for continuous suturing in LM. Even in cases of multiple myomas, the use of barbed suturing of only a single myomectomy wound appears to effectively reduce operation time by approximately 25%.

## Electronic supplementary material

Additional file 1: **Conventional intracorporeal suturing.** (ZIP 16 MB)

Additional file 2: **Passing the needle through the tail loop.** (ZIP 18 MB)

Additional file 3: **Long unidirectional barbed suturing technique with extracorporeal traction.** (ZIP 17 MB)

Additional file 4: **Baseball suturing.** (ZIP 17 MB)

Additional file 5: **Cutting the barbed suture thread.** (ZIP 11 MB)
